# The impact of individual cytochrome P450 enzymes on oxidative metabolism of benzo[*a*]pyrene in human livers

**DOI:** 10.1002/em.22001

**Published:** 2016-02-26

**Authors:** Miroslav Šulc, Radek Indra, Michaela Moserová, Heinz H. Schmeiser, Eva Frei, Volker M. Arlt, Marie Stiborová, P. White

**Affiliations:** ^1^ Department of BiochemistryFaculty of Science, Charles UniversityPragueCzech Republic; ^2^ Division of Radiopharmaceutical ChemistryGerman Cancer Research Center (DKFZ)HeidelbergGermany; ^3^ Analytical and Environmental Sciences DivisionMRC‐PHE Centre for Environment and Health, King's College LondonLondonUnited Kingdom; ^4^ NIHR Health Protection Research Unit in Health Impact of Environmental Hazards at King's College London in Partnership with Public Health EnglandLondonUnited Kingdom

**Keywords:** benzo[a]pyrene, cytochrome P450, metabolism, human liver

## Abstract

Benzo[*a*]pyrene (BaP) is a human carcinogen that covalently binds to DNA after metabolic activation by cytochrome P450 (CYP) enzymes. In this study human recombinant CYPs (CYP1A1, 1A2, 1B1, 2A6, 2B6, 2C8, 2C9, 2C19, 2E1, 3A4, and 3A5) were expressed in Supersomes™ together with their reductases, NADPH:CYP oxidoreductase, epoxide hydrolase and cytochrome *b_5_*, to investigate BaP metabolism. Human CYPs produced up to eight BaP metabolites. Among these, BaP‐7,8‐dihydrodiol and BaP‐9‐ol, which are intermediates in BaP‐derived DNA adduct formation, were mainly formed by CYP1A1 and 1B1, and to a lesser extent by CYP2C19 and 3A4. BaP‐3‐ol, a metabolite that is a ‘detoxified’ product of BaP, was formed by most human CYPs tested, although CYP1A1 and 1B1 produced it the most efficiently. Based on the amounts of the individual BaP metabolites formed by these CYPs and their expression levels in human liver, we determined their contributions to BaP metabolite formation in this organ. Our results indicate that hepatic CYP1A1 and CYP2C19 are most important in the activation of BaP to BaP‐7,8‐dihydrodiol, whereas CYP2C19, 3A4, and 1A1 are the major enzymes contributing to the formation of BaP‐9‐ol. BaP‐3‐ol is predominantly formed by hepatic CYP3A4, while CYP1A1 and 2C19 are less active. Environ. Mol. Mutagen. 57:229–235, 2016. © 2016 The Authors. Environmental and Molecular Mutagenesis Published by Wiley Periodicals, Inc.

## INTRODUCTION

Benzo[*a*]pyrene (BaP) is a polycyclic aromatic hydrocarbon (PAH) that has been classified as a human carcinogen (Group 1) by the International Agency for Research on Cancer [IARC, 2010]. BaP requires metabolic activation catalyzed by cytochrome P450 (CYP) enzymes prior to reaction with DNA [Baird et al., 2005]. The concentration of BaP in organisms is crucial for the induction of malignant transformations initiated by activated BaP. In addition to the total amount of ingested BaP, metabolism dictates its effective concentration, which thereby modulates BaP (geno)toxicity. BaP (geno)toxicity is also modulated by a number of confounding factors including other environmental pollutants (such as PAHs), human health status, diets, etc. [IARC, 2010]. Nevertheless, the identification of enzymes principally involved in BaP metabolism in humans and detailed knowledge of their catalytic specificities is of major importance.

CYP1A1 is one of the most important CYP enzymes in BaP bioactivation to species forming DNA adducts [Baird et al., 2005], in combination with microsomal epoxide hydrolase (mEH). First, CYP1A1 oxidizes BaP to an epoxide, which is then converted to BaP‐7,8‐dihydrodiol by mEH. Further bioactivation by CYP1A1 leads to the ultimately reactive species, BaP‐7,8‐dihydrodiol‐9,10‐epoxide (BPDE) that can react with DNA to form adducts preferentially at guanine residues (Supporting Information Fig. 1). The major product of the reaction of BPDE with DNA in vitro and in vivo is the adduct 10‐(deoxyguanosin‐*N* ^2^‐yl)−7,8,9‐trihydroxy‐7,8,9,10‐tetrahydrobenzo[*a*]pyrene (dG‐*N* ^2^‐BPDE) [Bauer et al., 1995; Arlt et al., 2008, 2015; Kucab et al., 2015]. However, BaP is also oxidized to other metabolites such as other dihydrodiols, BaP‐diones and hydroxylated metabolites [Bauer et al., 1995; Kim et al., 1998; Baird et al., 2005; Jiang et al., 2007]. While most of these metabolites are detoxification products, BaP‐9‐ol is a precursor of 9‐hydroxy‐BaP‐4,5‐epoxide, which can also form an adduct with deoxyguanosine in DNA (Supporting Information Fig. 1) [Stiborova et al., 2014].

CYP1B1 also oxidizes BaP, although its efficiency is about half of that of CYP1A1 [Kim et al., 1998]. Among other CYP enzymes, CYP1A2, 2C8, 2C9, 2C19, and 3A4 have all been reported to oxidize BaP, but their efficiencies are more than one order of magnitude lower than CYP1A1 [Bauer et al., 1995; Kim et al., 1998; Baird et al., 2005]. However, little is known about the impact of individual CYP enzymes on BaP metabolism in human tissues, which is crucial to understanding the carcinogenicity of BaP [IARC, 2010]. Lung, skin, stomach, and to a lesser extent, liver, are targets for BaP carcinogenicity. These organs all contain enzymes that metabolize xenobiotics including BaP. In this study, we evaluated the contributions of individual CYP enzymes expressed in human liver to BaP metabolism. We utilized human recombinant CYPs expressed in microsomes of baculovirus‐infected insect cells (Supersomes™) with their reductase NADPH:CYP oxidoreductase (POR) and mEH in the presence and absence of cytochrome *b_5_*, a known modulator of enzymatic activity of several CYPs [Porter, 2002; Schenkman and Jansson, 2003; Stiborova et al., 2006; McLaughlin et al., 2010; Kotrbova et al., 2011; Henderson et al., 2013; Stiborova et al., 2014]. To identify the contributions of individual hepatic CYPs to BaP metabolism, the formation of BaP metabolites by individual human CYPs was determined by HPLC analysis and correlation with associated CYP enzyme expression levels in human livers was investigated.

## MATERIALS AND METHODS

### Chemicals

BaP (CAS no. 50‐32‐8; purity ≥96%) was obtained from Sigma Chemical Co (St. Louis, MO, USA).

### Isolation of Cytochrome *b_5_*

Cytochrome *b_5_* was isolated from rabbit liver microsomes as described previously [Stiborova et al., 2014] and used for the reconstitution experiments.

### Enzymatic Incubations

Supersomes™ are microsomes isolated from insect cells that have been transfected with a baculovirus construct containing cDNA of human CYPs (CYP1A1, 1A2, 1B1, 2A6, 2B6, 2C8, 2C9, 2C19, 2E1, 3A4, and 3A5), and which also express POR, mEH and/or cytochrome *b_5._* Supersomes™were purchased from Gentest Corp. (Woburn, MI, USA) and were used to study BaP oxidation. Incubation mixtures contained 100 mM potassium phosphate buffer (pH 7.4), NADPH‐generating system (1 mM NADP^+^, 10 mM d‐glucose‐6‐phosphate, 1 U/ml d‐glucose‐6‐phosphate dehydrogenase), 100 nM human CYPs in Supersomes™, and 50 μM BaP (dissolved in 5 μl dimethyl sulfoxide) in a final volume of 500 μl. As Supersomes™ expressing CYP1A1, 1A2, and 1B1 did not contain cytochrome *b_5_*, the supersomal system was reconstituted with purified cytochrome *b_5_*, isolated as described above. Enzyme reconstitution utilizing these CYP systems in Supersomes™ and purified cytochrome *b_5_* was performed as described elsewhere [Stiborova et al., 2002, 2005, 2006; Kotrbova et al., 2011; Indra et al., 2014; Stiborova et al. 2014], using a molar ratio of CYPs to cytochrome *b_5_* of 1:5. The reaction was initiated by adding 50 μl of the NADPH‐generating system. Control incubations were carried out either without the enzymatic system (the CYP systems), or without the NADPH‐generating system, or without BaP. After incubation (37°C, 20 min), 5 μl of 1 mM phenacetin (Sigma) in methanol was added as an internal standard. BaP metabolites were extracted twice with ethyl acetate (2 × 1 ml), after which the solvent was evaporated to dryness, with the remaining residues dissolved in 25 μl methanol, followed by separation of BaP metabolites by HPLC. BaP metabolite peaks were collected and analyzed by NMR and/or mass spectrometry [Stiborova et al., 2014]. Further details on the methods are given in Supporting Information.

### HPLC Analysis of BaP Metabolites

HPLC analysis of BaP metabolites was carried out as described [Stiborova et al., 2014]. BaP metabolite peaks (Supporting Information Fig. 2) were collected and analyzed by NMR and/or mass spectrometry as reported [Stiborova et al., 2014]. The peak areas at 254 nm were calculated relative to the peak area of the internal standard phenacetin, and expressed as relative peak areas.

### Contributions of Human CYP Enzymes to the Formation of BaP‐7,8‐Dihydrodiol, BaP‐9‐Ol, and BaP‐3‐Ol in Human Liver

In order to calculate the contributions of individual CYPs to the formation of BaP‐7,8‐dihydrodiol, BaP‐9‐ol, and BaP‐3‐ol in human livers, we measured the velocities of their formation by the Supersomal CYP enzyme systems containing cytochrome *b_5_* (compare Fig. 2), and combined these velocities with data on the average expression levels of individual CYPs in human livers derived from our earlier studies on CYP1A1 [Stiborova et al., 2002, 2005], or from Rendic and Di Carlo [1997]. Specifically, the contributions of each CYP to BaP metabolite formation in liver were calculated by dividing the relative metabolite‐forming activity of each CYP [r.a._cypi_] (rate of formation of BaP‐7,8‐dihydrodiol, BaP‐9‐ol, and BaP‐3‐ol multiplied by amounts of this CYP in human liver) by the total relative activities (**∑**[r.a._cypi_]) of all metabolite‐forming CYPs. CYP3A4 is the most highly expressed CYP in human liver (∼30% of the CYP hepatic complement), followed by CYP2C9 and 1A2 (∼15 and ∼13%, respectively), while CYP2C19, 2E1 2A6, 2D6, 2C8, and 3A5 each represent between ∼8.5 and ∼2.5% of liver CYPs [Rendic and Di Carlo, 1997]. Finally, a low but detectable amount of CYP2B6 is also expressed in human liver (∼0.2% of liver CYPs). CYP1A1 and particularly CYP1B1 are both considered to be extrahepatic CYP enzymes, although their expression in human liver can be induced by various chemicals, resulting in them constituting <0.7% and <0.1% of the liver CYP complement, respectively [Rendic and DiCarlo, 1997; Stiborova et al., 2002, 2005].

## RESULTS AND DISCUSSION

### Oxidation of BaP by Human Recombinant CYPs in Supersomes™

The metabolism of PAHs has been intensively studied over the past decades [Baird et al., 2005], and various studies have examined the roles of individual human CYPs (particularly CYP1A1 and CYP1B1) to metabolize BaP in several enzyme systems [Bauer et al., 1995; Kim et al., 1998; Baird et al., 2005; Indra et al., 2014; Stiborova et al., 2014]. However, previous studies often used CYP systems that do not fully correspond to the natural enzyme system located in the membrane of the endoplasmic reticulum (microsomes), as cytochrome *b_5_* (a known modulator of enzymatic activity of several CYPs [Porter, 2002; Schenkman and Jansson, 2003; Stiborova et al., 2006; Kotrbova et al., 2011; McLaughlin et al., 2010; Stiborova et al., 2014]) was not incorporated. In order to better model hepatic microsomes, we utilized enzyme systems containing microsomes (Supersomes™) together with human CYPs (CYP1A1, 1A2, 1B1, 2A6, 2B6, 2C8, 2C9, 2C19, 2E1, 3A4, and 3A5), POR, mEH, and cytochrome *b_5_*. Cytochrome *b_5_* was either expressed in Supersomes™ together with CYPs, POR, and mEH, or Supersomes™ were reconstituted with purified cytochrome *b_5_*. These CYP enzyme systems efficiently oxidized their typical substrates (data not shown). The BaP metabolite profile formed by individual human CYPs in Supersomes™ was determined by HPLC analysis, and BaP metabolites were identified by NMR and/or mass spectrometry as described previously [Stiborova et al., 2014].

**Figure 1:**
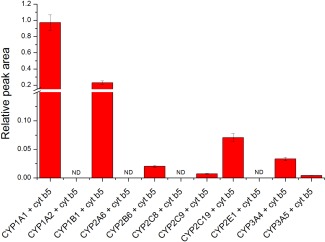
BaP metabolism catalyzed by individual human CYPs expressed in Supersomes™ in the presence of cytochrome *b_5_* (*b*_5_). Columns show the sum of individual BaP metabolites formed by CYPs. Data shown are averages and standard deviation from three independent measurements. ND, not detected.

**Figure 2:**
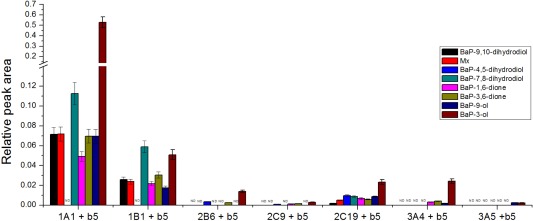
Formation of BaP metabolites by human CYPs expressed in Supersomes™ in the presence of cytochrome *b_5_* (*b*_5_). Data shown are averages and standard deviation from three independent measurements. ND, not detected.

Up to eight BaP metabolites were separated by HPLC using the CYP enzyme systems (Supporting Information Fig. 2): BaP‐9,10‐dihydrodiol (M1), BaP‐4,5‐dihydrodiol (M2), BaP‐7,8‐dihydrodiol (M3), BaP‐1,6‐dione (M4), BaP‐3,6‐dione (M5), BaP‐9‐ol (M6), BaP‐3‐ol (M7), and a metabolite of unknown structure (Mx). When the NADPH‐generating system was omitted from the incubation mixtures, essentially no BaP metabolites were formed (data not shown). Amounts of the various BaP metabolites formed were dependent on the individual CYP isoenzyme studied (Figs. 1 and 2).

Of the human recombinant CYPs tested in these enzymatic systems, CYP1A2, 2A6, 2C8, and 2E1 did not catalyze BaP oxidation (Fig. 1). It should be noted that contrasting results have been found with regards to the role of human CYP1A2 in BaP oxidation. Whereas some studies found that human CYP1A2 partially oxidized BaP [Bauer et al. 1995; Kim et al. 1997], other studies reported that human CYP1A2 is not effective in mediating BaP hydroxylation [Endo et al. 2008]. In our system, CYP1A1 had the highest BaP‐metabolizing potency, followed by CYP1B1, 2C19, 3A4, 2B6, 2C9, and 3A5 (Fig. 1).

Of the BaP metabolites generated by the CYP enzyme systems, BaP‐3‐ol was formed by most of the CYPs active in BaP metabolism as the major BaP oxidation product, except CYP1B1 (Fig. 2). This BaP oxidation product is considered to be a detoxification metabolite [Huang et al., 1996]. In addition, BaP‐7,8‐dihydrodiol and BaP‐9‐ol were formed (Fig. 2), which are precursors of BPDE and 9‐hydroxy‐BaP‐4,5‐oxide respectively, both of which covalently bind to DNA forming adducts (see Supporting Information Fig. 1). Of the CYPs tested here, CYP1A1, 1B1, and CYP2C19 were capable of forming BaP‐7,8‐dihydrodiol (Fig. 2). The formation of BaP‐9‐ol was also mainly catalyzed by CYP1A1 and 1B1, while CYP2C19 and CYP3A produced this metabolite to a lesser extent (Fig. 2). CYP1A1 and 1B1 were also the most effective in catalyzing oxidation of BaP to BaP‐3‐ol, although this metabolite was also formed by CYP2B6, 2C9, 2C19, and 3A4/5 (Fig. 2).

**Figure 3:**
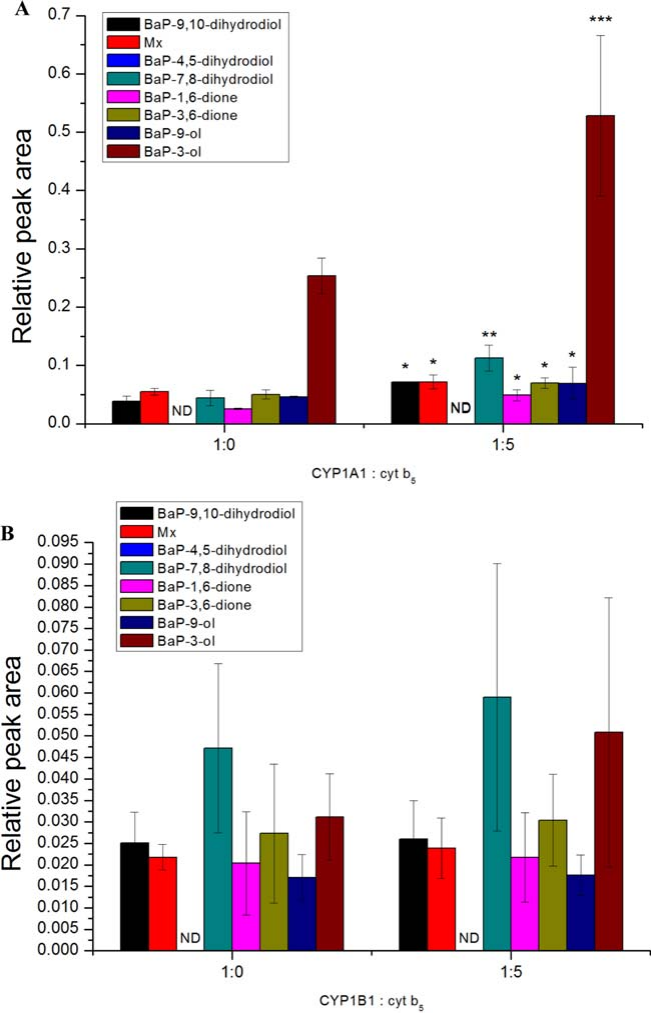
Oxidation of BaP by human recombinant CYP1A1 (**A**) and CYP1B1 (**B**) expressed in Supersomes™ and the effect of cytochrome *b_5_* (cyt *b*_5_) on this oxidation. Data shown are averages and standard deviation from three independent measurements. ND, not detected. Comparison was performed by Student *t*‐test analysis; *P <* *0.05, **P <* *0.01, ***P <* *0.001, different from CYP1A1‐mediated oxidation of BaP without cytochrome *b_5_*.

Interestingly, we found that cytochrome *b_5_* strongly modulated the activity of some CYPs to metabolize BaP, most notably CYP1A1 (Fig. 3). The CYP1A1 enzyme system generated the largest amount of BaP‐3‐ol (Fig. 3A). Addition of cytochrome *b_5_* to CYP1A1 in Supersomes™ increased BaP oxidation to this metabolite more than twofold. The greatest stimulatory effect of cytochrome *b_5_* was in the formation of BaP‐3‐ol and BaP‐7,8‐dihydrodiol, and to a lesser extent on the other BaP metabolites produced (Fig. 3A). Overall, in the enzyme system used, human CYP1A1 metabolized BaP to seven metabolites: BaP‐7,8‐dihydrodiol, BaP‐9,10‐dihydrodiol, BaP‐1,6‐dione, BaP‐3,6‐dione, BaP‐3‐ol, BaP‐9‐ol, and the metabolite Mx (Figs. 2 and 3A). In contrast, BaP‐4,5‐dihydrodiol was not detected in the CYP1A1 system (Figs. 2 and 3A) which is consistent with previous findings showing that this BaP metabolite was also not formed in human bronchoalveolar H358 cells expressing CYP1A1 exposed to BaP [Jiang et al., 2007].

Sudan I and 7‐ethoxyresorufin are both used as markers for CYP1A enzyme activity. Interestingly, cytochrome *b_5_* has been previously shown to stimulate CYP1A1‐mediated oxidation of Sudan I [Stiborova et al., 2002, 2005, 2006] but not 7‐ethoxyresorufin [Stiborova et al., 2005, 2006]. Similarly, we have previously shown that cytochrome *b_5_* impacts CYP1A1‐mediated metabolism of the anticancer drug ellipticine [Kotrbova et al., 2011]. Two mechanisms have been suggested to describe how cytochrome *b_5_* modulates CYP catalysis: 1) it can affect CYP catalytic activities by donating the second electron to CYP in a CYP catalytic cycle; and/or 2) it can act as an allosteric modifier of the oxygenase [Porter, 2002; Schenkman and Jansson, 2003; Guengerich, 2005; Kotrbova et al., 2011]. However, the mechanism(s) underlying such allosteric effects, based on reports that apo‐cytochrome *b_5_* can stimulate CYP catalysis, are still uncertain at present. Nevertheless, it seems to be clear that cytochrome *b*_5_ binding can cause conformational changes to the substrate access channel and binding pocket in the CYP enzyme [Porter, 2002; Estrada et al., 2014]. Addition of cytochrome *b_5_* to the CYP enzyme system changed both the absolute amounts, and to a lesser extent the relative amounts, of the individual BaP metabolites formed by CYP1A1 (Fig. 3A). Thus, interaction of CYP1A1 with cytochrome *b_5_* could result both in conformational changes in the CYP1A1 protein molecule and/or an impact on the electron transfer from cytochrome *b_5_* to CYP1A1, thereby providing mechanisms to explain the observed increase in BaP oxidation. Nevertheless, further investigations are required to clarify the mechanism responsible for the effects of cytochrome *b_5_* on CYP1A1‐mediated oxidation of BaP.

Human CYP1B1 oxidized BaP to the same metabolites as CYP1A1 (Figs. 2 and 3B). As with CYP1A1, BaP‐4,5‐dihydrodiol was not been detected in the CYP1B1 enzyme system. BaP‐7,8‐dihydrodiol was the major BaP metabolite generated by CYP1B1. However, in contrast to CYP1A1, addition of cytochrome *b_5_* to the CYP1B1 enzyme system had no significant effect on BaP oxidation (Fig. 3B).

### Contributions of Individual CYPs to BaP Metabolism in Human Liver

BaP‐7,8‐dihydrodiol, BaP‐9‐ol and BaP‐3‐ol are critical intermediates that contribute to the (geno)toxicity of BaP. Therefore, it is crucial to examine which of the CYP enzymes expressed in human liver are most important for the formation of these metabolites. Based on the amounts of BaP‐7,8‐dihydrodiol, BaP‐9‐ol and BaP‐3‐ol formed by each human CYP enzyme, and the expression levels of these enzymes in human liver [Rendic and DiCarlo, 1997; Stiborova et al., 2002, 2005], we estimated the contributions of individual CYPs to the formation of these BaP metabolites in human liver.

**Figure 4:**
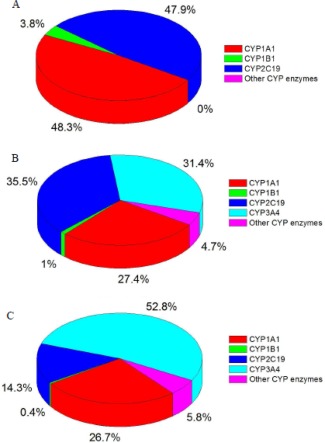
Contributions of CYP enzymes to the formation BaP‐7,8‐dihydrodiol (**A**), BaP‐9‐ol (**B**), and BaP‐3‐ol (**C**) in human livers.

Although CYP1A1 is expressed in human liver at very low levels (<0.7%) [Rendic and DiCarlo, 1997; Stiborova et al., 2002, 2005], it is estimated to form more BaP‐7,8‐dihydrodiol than any other human CYP (∼48.3% of all BaP‐7,8‐dihydrodiol synthesis) (Fig. 4A). A similar contribution to the formation of BaP‐7,8‐dihydrodiol is attributed to CYP2C19 (∼47.9%), whereas CYP1B1 contributed little (∼3.8%) to its formation (Fig. 4A). Other human hepatic CYPs have essentially no impact on the formation of this metabolite.

For BaP‐9‐ol, the highest contribution to its formation is attributed to CYP2C19 (∼35.5%), followed by CYP3A4 (∼31.4%), and CYP1A1 (∼27.4%); in contrast, CYP3A5 (∼4.7%) and CYP1B1 (∼1%) had minor impacts on its formation (Fig. 4B). In human liver, the formation of BaP‐3‐ol, which is the major metabolite formed by most of the tested human CYP enzymes, is attributed to CYP3A4 (∼52.8%), followed by CYP1A1 (∼26.7%) and CYP2C19 (∼14.3%). CYP2C9 (∼3.3%), 2B6 (∼2.1%), 3A5 (∼0.4%), and 1B1 (∼0.4%) also participate in the formation of this detoxification BaP metabolite in human liver, but their contribution is minor (Fig. 4C). Other CYP enzymes expressed in human liver have essentially no ability to form BaP‐3‐ol.

## CONCLUSIONS

In the present study, we identified the human CYPs expressed in a microsomal system (i.e. Supersomes™) that are most effective in BaP metabolism in the presence of POR, mEH, and cytochrome *b_5_*. We showed that BaP is metabolized to up to eight metabolites including the activation metabolites BaP‐7,8‐dihydrodiol and BaP‐9‐ol, and the detoxification metabolite BaP‐3‐ol. We showed that BaP‐7,8‐dihydrodiol and BaP‐9‐ol are mainly formed by CYP1A1 and 1B1, and to a lesser extent by CYP2C19 and CYP3A4. In contrast, the formation of BaP‐3‐ol is most efficiently catalyzed by CYP1A1 and 1B1, but CYP2B6, 2C9, 2C19, and 3A4 also partially contribute to its production.

Based on amounts of BaP‐7,8‐dihydrodiol, BaP‐3‐ol, and BaP‐9‐ol formed by individual human CYP enzymes, and expression levels of CYPs in human liver, we determined the contributions of CYPs to their formation in this human organ. However, it should be noted that actual hepatic concentrations are also influenced by the phase II enzymes [Shi et al., 2015] that were not evaluated in the present study. In human liver CYP1A1 and CYP2C19 are most important in the activation of BaP to BaP‐7,8‐dihydrodiol, producing ∼48% of the total amount of this metabolite; whereas, CYP2C19 (35%), 3A4 (31%), and 1A1 (26%) are the major enzymes contributing to the formation of BaP‐9‐ol. The detoxification of BaP to BaP‐3‐ol is predominantly catalyzed by hepatic CYP3A4, the major CYP enzyme expressed in human livers, contributing to more than 50% of its formation, while CYP1A1 (27%) and 2C19 (14%) contribute to a lesser extent.

Our study demonstrates that the degree of activation or detoxification of BaP in human liver is affected by both the activities of individual human CYPs to metabolize BaP, and the expression levels of these CYP enzymes in human. Therefore, modulation of levels and activities of hepatic CYPs, mediated both by their polymorphisms (or internal regulation), and their induction or inhibition by endogenous and exogenous compounds, determines the (geno)toxic properties of BaP. Approaches analogous to those carried out in the present study might be utilized for evaluating the contributions of human CYPs to BaP metabolism in the lung, a target organ for BaP carcinogenicity. However, as CYP expression levels in the human lung have not yet been fully characterized, this remains a challenge for future studies.

## AUTHOR CONTRIBUTIONS

M.S., E.F., H.H.S., and V.M.A. designed the study. M.Š., R.I., and M.M. performed the experiments, analyzed the data, and prepared the figures. M.S. and V.M.A. prepared the manuscript draft and had complete access to the study data. All authors approved the final manuscript.

## Supporting information

Supporting Information
