# Supplementary material for: The impact of individual cytochrome P450 enzymes on oxidative metabolism of benzo[a]pyrene in human livers
Source: Environ Mol Mutagen. 2016 Feb 26;57(3):229–35. doi: 10.1002/em.22001 (PMC4855618; doi:10.1002/em.22001)
Supplement: Supplementary file 1 — Supporting Information [file EM-57-229-s001.doc]

**Supporting Information**

**The Impact of Individual Cytochromes P450 Enzymes on the Oxidation Metabolism of Benzo[*a*]pyrene in Human Livers**

**Miroslav Šulc,^1^ Radek Indra,^1^ Michaela Moserová,^1^ Heinz H. Schmeiser,^2^ Eva Frei,^1^ Volker M. Arlt,^3,4^ and Marie Stiborová^1*^**


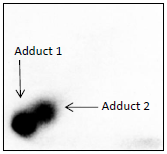

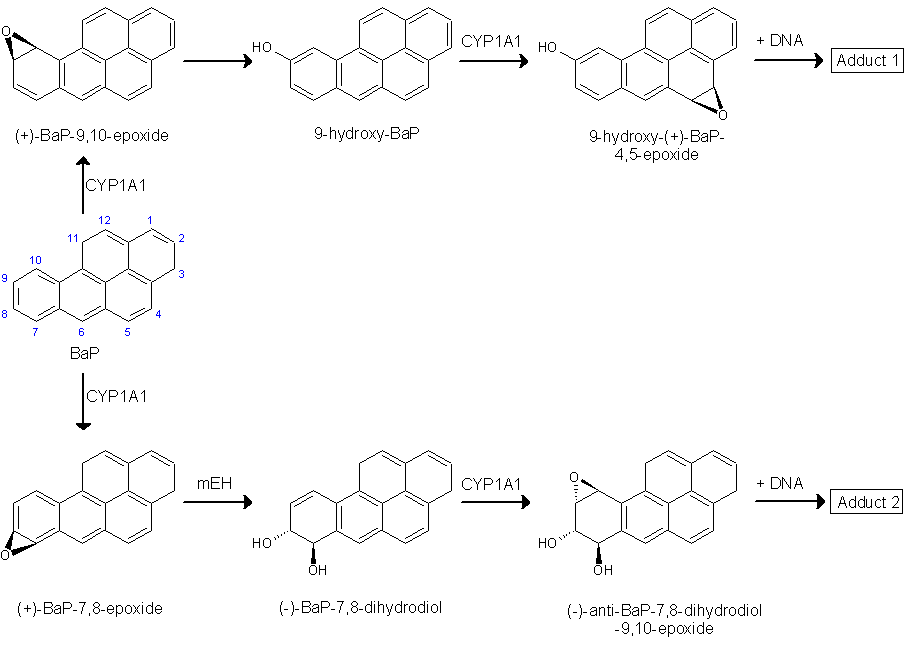


**Supporting Fig. 1** Proposed pathways of biotransformation and DNA adduct formation of BaP catalyzed by CYP1A1 and mEH. As shown in the upper part of the figure, the two-step activation process by CYP1A1 leads to the formation of 9-hydroxy-BaP-4,5-epoxide that can react with deoxyguanosine in DNA (adduct 1). As shown in the lower part of the figure, the typical three-step activation process by CYP1A1 followed by hydrolysis by mEH leads to BPDE which forms dG-*N*^2^-BPDE (adduct 2). Insert: Autoradiographic profile of BaP-DNA adducts formed by CYP1A1 with mEH as evaluated by thin-layer chromatography ^32^P-postlabeling as described previously [Stiborova et al., 2014]; the arrows show adducts 1 and 2.

**A**

M1

Mx

M3

M4

M5

M6

M7

BaP

**B**


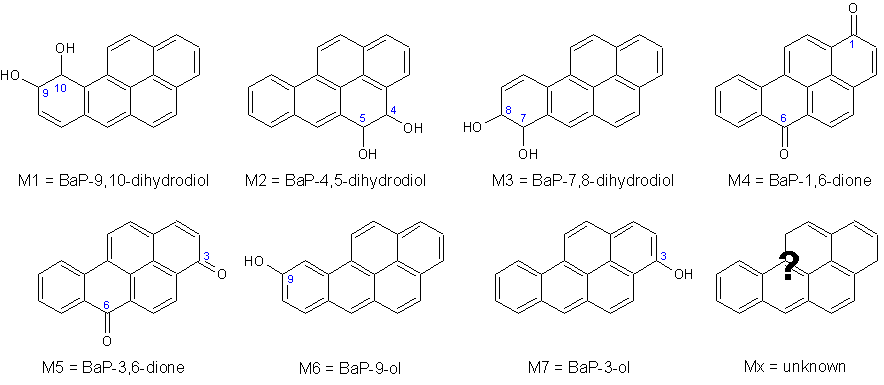


**Supporting Fig. 2**. HPLC analysis of BaP metabolites formed by human recombinant CYP1A1 expressed in Supersomes™ (**A**). Structures of BaP metabolites M1-M7 formed by human CYPs (**B**).

**Supporting information on NMR and mass spectra of BaP metabolites** [Stiborova et al., Toxicology 318:1-12, 2014]

**NMR spectrometry**

NMR spectra (δ, ppm; *J*, Hz) of BaP and its metabolites M1, M4, M5 and M7 were measured on a Bruker Avance II-600 and/or Bruker Avance II-500 instruments equipped with a cryoprobe (600.1 or 500.0 MHz for ^1^H and 150.9 or 125.7 MHz for ^13^C) in hexadeuterated acetone and CDCl_3_ and referenced to the solvent signals (δ 2.05 and 7.27, respectively). Due to the low amounts of metabolites it was not possible to acquire ^13^C NMR spectra or perform heteronuclear correlation experiments.

1) BaP. ^1^H NMR spectrum of BaP contains five isolated spin systems: one three-spin system of hydrogens H-1, H-2 and H-3, one four-spin system of H-7, H-8, H-9 and H-10, one isolated spin of H-6 and two two-spin systems of H-4, H-5 and of H-11, H-12. The assignment of signals in the spectrum could be done with the help of homonuclear COSY spectrum, where strong cross-peaks corresponding to three-bond couplings were observed. However, the signals of the two two-spin systems (H-4,H-5 and H-11, H-12) could not be assigned with the use of COSY spectrum only and heteronuclear correlation spectra (HSQC and HMBC) had to be used. The assignment of all ^1^H signals and coupling constants of BaP and its metabolites is shown in Supporting Table 1 (for structures see Supporting Fig. 2B).

2) M1. Two signals of the four-spin system of M1 were shifted upfield (to 4.5 and 5.7 ppm). These values are too low for the fully aromatic BaP skeleton. The structure of M1 was identified as *trans*-9,10-dihydro-BaP-9,10-diol (BaP-9,10-dihydrodiol). The *trans* arrangement of the two hydroxy groups was supported by the inspection of the vicinal coupling constant between hydrogen atoms H-9 and H-10. The torsion angle between these two hydrogen atoms calculated using the generalized Karplus type equation [Haasnoot et al., Tetrahedron 36:2783-2792, 1980] was predicted to be 48º. This value is very close to the torsion angle observed in the molecular model of the *trans*-derivative with the two hydroxyl groups in pseudo-axial positions. The molecular model of the *cis*-derivative predicts the torsion angle between the two hydrogen atoms to be close to −75º. We were unable to determine the absolute configuration on the new asymmetric centers (C-9 and C-10). It is possible that both enantiomers are present in metabolite M1.

3) M4 and M5. In the spectra of both compounds we observed the four-spin system of H-7, H-8, H-9 and H-10 and three two-spin systems. Two substituents are therefore attached to the BaP skeleton: one in position 6 and the second one in position 1 or 3. Furthermore, the unusually shielded H-2 proton at 6.7 ppm was characteristic of the α-proton (next to carbonyl) in phenalones suggesting that the structures of M4 and M5 could be BaP-1,6-dione and -3,6-dione, respectively.

4) M7. In the spectrum of M7, the three-spin system was replaced with a two-spin system suggesting that one substituent is attached to the position 1 or 3 of the BaP. The structure of M7 was confirmed to be BaP-3-ol by comparison of the chemical shifts and coupling constants with those of BaP-3-ol standard.

5) M2, M3 and M6. Because the amounts of M2, M3 and M6 samples were insufficient for NMR spectroscopy, these metabolites were analyzed only by mass spectrometry only as described below.

**Mass spectrometry**

Mass spectra of BaP and its metabolites M2, M3 and M6 were measured on a matrix-assisted laser desorption/ionisation reflectron time-of-flight MALDITOF mass spectrometer ultraFLEX III (Bruker-Daltonics, Bremen, Germany). Positive spectra were calibrated externally using the monoisotopic [M+H]^+^ ion of MRFA peptide 524.26 *m/z* and CCA matrix peaks 190.05, 379.09 *m/z*. A 10 mg/ml solution of α-cyano-4-hydroxy-cinnamic acid or 2,5-dihydrobenzoic acid in 50% MeCN/0.3% acetic acid was used as MALDI matrix. A 0.5 μl of sample dissolved in MeCN was premixed with 0.5 μl of the matrix solution on the target and allowed to dry at ambient temperature. The MALDI-TOF positive spectra were collected in reflectron mode. Positive [M^+^] of BaP corresponded to this compound (*m/z* 252.1). The metabolites with retention times of 11.9 (M2) and 12.9 min (M3) gave a positive molecular ion each at *m*/*z* 286.1 which is indicative of BaP-dihydrodiol metabolites. The metabolite eluted at 24.6 min (M6) gave a positive molecular ion at *m/z* 268.1 which is indicative of a hydroxylated BaP metabolite. These results are consistent with previous studies on the metabolism of BaP by human CYP1A1 [Bauer et al., Chem Res Toxicol 8:136-142, 1995; Kim et al., Carcinogenesis 19:1847-1853, 1998], in which these metabolites were identified as BaP-4,5-dihydrodiol (M2), BaP-7,8-dihydrodiol (M3), and BaP-9-ol (M6).

**Supporting Table** ^1^H chemical shifts of BaP and its metabolites M1, M4, M5 and M7 in hexadeuterated acetone. Coupling constants are in parenthesis.

|  | **BaP** | **M1** | **M4** | **M5** | **M7** |
| --- | --- | --- | --- | --- | --- |
| H-1 | 8.33 d  (*7.8*) | 8.26^a^ d  (*7.6*) | - | 8.02 d  (*9.7*) | 8.20 d  (*8.5*) |
| H-2 | 8.04 t  (*7.6*) | 8.04 t  (*7.6*) | 6.74 d  (9.8) | 6.69 d  (*9.7*) | 7.68 d  (*8.4*) |
| H-3 | 8.18 d  (*7.4*) | 8.27^a^ d  (*7.6*) | 8.03 d  (9.7) | - | - |
| H-4 | 8.09 d  (*9.2*) | 8.12^b^ d  (*8.9*) | 8.19 d  (7.4) | 8.83^a^ d  (*7.7*) | 8.30 d  (*9.3*) |
| H-5 | 8.01 d  (*9.1*) | 8.15^b^ d  (*8.9*) | 8.68 d  (7.4) | 8.71^a^ d  (*7.7*) | 8.00 d  (*9.5*) |
| H-6 | 8.65 s | 8.09 s | - | - | 8.52 s |
| H-7 | 8.37 dm  (*8.1*) | 7.02 d  (*9.5*) | 8.41 dm  (7.7) | 8.41 dm  (*7.9*) | 8.32 s  (*8.1*) |
| H-8 | 7.83 ddd  (*8.0, 6.7, 1.2*) | 6.38 dd  (*9.6, 5.3*) | 7.73 m | 7.70 m | 7.79 m |
| H-9 | 7.88 ddd  (*8.4, 6.7, 1.4*) | 4.50 dd  (*5.2, 1.7*) | 7.92 m | 7.90 m | 7.81 m |
| H-10 | 9.18 dm  (*8.5*) | 5.69 m | 8.66 dm  (8.0) | 8.61 dm  (*8.1*) | 9.12 d  (*8.3*) |
| H-11 | 9.20 d  (*9.2*) | 8.58 d  (*9.2*) | 8.60^a^ d  (7.9) | 8.76 d  (*7.7*) | 8.97 d  (*9.1*) |
| H-12 | 8.43 d  (*9.1*) | 8.23 d  (*9.3*) | 8.92^a^ d  (7.9) | 8.12 d  (*7.7*) | 8.30 d  (*9.3*) |

^a,b^Chemical shifts with the same letter in one column may be interchanged.
